# Development and Validation of a Predictive Model Based on Serum Silent Information Regulator 6 Levels in Chinese Older Adult Patients: Cross-Sectional Descriptive Study

**DOI:** 10.2196/64374

**Published:** 2025-01-15

**Authors:** Yuzi You, Wei Liang, Yajie Zhao

**Affiliations:** 1 Department of General Practice, Ruijin Hospital, Shanghai Jiao Tong University School of Medicine, Shanghai, China; 2 Department of Geriatrics, Ruijin Hospital, Shanghai Jiao Tong University School of Medicine, 197 Ruijin Er Road, Shanghai, 200025, China, 86 13601893105

**Keywords:** aging, coronary artery disease, nomogram, SIRT6, TyG index, silent information regulator 6, triglyceride glucose index

## Abstract

**Background:**

Serum levels of silent information regulator 6 (SIRT6), a key biomarker of aging, were identified as a predictor of coronary artery disease (CAD), but whether SIRT6 can distinguish severity of coronary artery lesions in older adult patients is unknown.

**Objectives:**

This study developed a nomogram to demonstrate the functionality of SIRT6 in assessing severity of coronary artery atherosclerosis.

**Methods:**

Patients aged 60 years and older with angina pectoris were screened for this single-center clinical study between October 1, 2022, and March 31, 2023. Serum specimens of eligible patients were collected for SIRT6 detection by enzyme-linked immunosorbent assay. Clinical data and putative predictors, including 29 physiological characteristics, biochemical parameters, carotid artery ultrasonographic results, and complete coronary angiography findings, were evaluated, with CAD diagnosis as the primary outcome. The nomogram was derived from the Extreme Gradient Boosting (XGBoost) model, with logistic regression for variable selection. Model performance was assessed by examining discrimination, calibration, and clinical use separately. A 10-fold cross-validation technique was used to compare all models. The models’ performance was further evaluated on the internal validation set to ensure that the obtained results were not due to overoptimization.

**Results:**

Eligible patients (n=222) were divided into 2 cohorts: the development cohort (n=178) and the validation cohort (n=44). Serum SIRT6 levels were identified as both an independent risk factor and a predictor for CAD in older adults. The area under the receiver operating characteristic curve (AUROC) was 0.725 (95% CI 0.653‐0.797). The optimal cutoff value of SIRT6 for predicting CAD was 546.384 pg/mL. Predictors included in this nomogram were serum SIRT6 levels, triglyceride glucose (TyG) index, and apolipoprotein B. The model achieved an AUROC of 0.956 (95% CI 0.928‐0.983) in the development cohort. Similarly, in the internal validation cohort, the AUROC was 0.913 (95% CI 0.828‐0.999). All models demonstrated satisfactory calibration, with predicted outcomes closely aligning with actual results.

**Conclusions:**

SIRT6 shows promise in predicting CAD, with enhanced predictive abilities when combined with the TyG index. In clinical settings, monitoring fluctuations in SIRT6 and TyG may offer valuable insights for early CAD detection. The nomogram for CAD outcome prediction in older adult patients with angina pectoris may aid in clinical trial design and personalized clinical decision-making, particularly in institutions where SIRT6 is being explored as a biomarker for aging or cardiovascular health.

## Introduction

The global burden of ischemic heart disease from 1990 to 2019 reached staggering figures, with an estimated 9.14 million deaths attributed to coronary artery disease (CAD) in 2019 alone, affecting approximately 197 million individuals worldwide [1]. China witnessed a notable surge in CAD-associated mortality during this period, accounting for 38.2% of the global rise. By 2017, CAD-related deaths in China had skyrocketed by 1.12 million, representing an astonishing 184.1% increase compared with that of 1990 [2,3]. As of 2019, China was the global leader in CAD-related deaths, with 1.87 million reported cases [4]. By 2029, a 64% increase in CAD cases is expected in China compared with 2020. As a dynamically evolving condition, CAD significantly contributes to the development of major adverse cardiovascular events, such as myocardial infarction, stroke, and cardiovascular mortality [5].

Aging, an independent risk factor for CAD, is characterized by a progressive decline in coronary artery and microvascular function, resulting in altered myocardial perfusion and increased myocardial injury [6]. CAD predominantly affects middle-aged and older adult populations, particularly people aged 60 years and older, with a prevalence exceeding 70%. Notably, the older adult population in China, aged 65 years and older, reached 220 million in 2023, constituting 15.4% of the nation’s population and approximately 26.8% of the global older adult population [7]. The aging demographic in China is expanding rapidly, with nearly 40 million individuals aged 80 years and older in 2022, accounting for approximately 2.7% of the total population of China. Hence, China is predicted to enter a super-aged society by 2030, with the older adult population comprising more than 20% of the total population [8]. By 2084, it is estimated that half of China’s population will be older adults. A higher mortality rate has been observed among Chinese older adults with CAD, with the majority of deaths occurring in people aged 75 years and older [9]. Despite these trends, research on CAD has often overlooked the specific needs of the older adult population. In China, approximately 300 million people suffer from chronic diseases, with half of them being aged 65 years or older. The older adult population often presents with atypical clinical symptoms, multiple comorbidities, and prolonged use of medications, all of which amplify the risk of cardiovascular disease [10,11]. Previous studies have indicated that therapeutic interventions in the early stages of CAD can reduce its incidence and improve prognosis [12,13], underscoring the necessity for noninvasive methods for identifying predictive factors for CAD.

Silent information regulator 6 (SIRT6), a member of the nicotinamide adenine dinucleotide–dependent histone deacetylase family, plays a crucial role in aging and aging-related diseases by maintaining telomerase stability and metabolic homeostasis, as well as regulating oxidative stress [14]. Recent evidence has highlighted the protective properties of SIRT6 against CAD in preserving endothelial function, inhibiting inflammatory responses, and regulating glucose and lipid metabolism. Hence, a decline in serum SIRT6 levels has emerged as an independent risk factor for CAD [15], warranting further investigation of its diagnostic and predictive value in older adult patients. Existing risk assessment models, such as the Pooled Cohort Equations model and the Systematic Coronary Risk Estimation model, were primarily developed based on Caucasian and African American populations and lack specific biomarkers and demonstrate poor calibration when applied to the Chinese population [16]. Given these ethnic differences, there is an urgent need for tailored risk assessment models in cardiovascular disease prevention, particularly in diverse populations such as China.

In this study, we aimed to develop a nomogram for predicting outcomes in older adults presenting with clinical symptoms suggestive of CAD. We hypothesized that a combination of baseline SIRT6 levels with clinical parameters could improve the evidence-based selection of candidates for this marker and facilitate clinical decision-making, resulting in its potential implementation in clinical trials.

## Methods

### Study Design and Participants

In this single-center study, a nomogram was developed for predicting the outcomes of potential cases with CAD and was validated using data from Ruijin Hospital, Shanghai Jiao Tong University School Of Medicine. Patients aged 60 years or older who were diagnosed with angina pectoris were screened between October 1, 2022, and March 31, 2023. The Judkins method [17] was used for performing coronary angiography (CAG) via the radial or femoral artery. The angiographic results underwent joint assessment by 3 experienced cardiovascular specialists (WFS , ZBZ, and JWN). The severity of stenosis in the major coronary arteries, including the left anterior descending branch (LAD), left circumflex artery, right coronary artery, and left main coronary artery, was evaluated, with CAD defined as ≥50% stenosis in any 1 vessel and coronary atherosclerosis (CAS) as <50% stenosis in all vessels.

Eligible participants were individuals aged 60 years or older who presented with chest pain and had been evaluated by a specialist, resulting in a preliminary diagnosis of CAD with an indication for CAG. Participants must have no prior history of CAG, percutaneous coronary intervention, or coronary artery bypass grafting. Furthermore, informed consent must be obtained for the collection of biological samples, with blood samples obtained prior to CAG. Participants must also have coronary artery stenosis as confirmed by the CAG results. Exclusion criteria included recent acute infections, gastrointestinal bleeding, surgical procedures, or trauma within the past 6 months. Individuals were also excluded if they had positive viral markers, including hepatitis B surface antigen, hepatitis B core antibody with hepatitis B virus–DNA of the detection threshold or greater, positive hepatitis C virus antibody with hepatitis C virus–RNA of the upper limit of normal or greater, or positive HIV antibody. Severe cardiac conditions also warranted exclusion, including decompensated heart failure, significant valvular heart disease within the past 6 months, notable electrocardiographic abnormalities (eg, any degree of atrial fibrillation, second-degree type II or third-degree atrioventricular block, or corrected QT interval exceeding 470 milliseconds in females or 450 milliseconds in males), uncontrolled symptomatic arrhythmias, cerebrovascular events or transient ischemic attacks within the past 6 months, a history of malignancy or autoimmune diseases, or severe liver or renal disorders unrelated to the study condition.

Upon admission, patients were assessed for CAD severity using the Gensini score, an angiographic tool for grading coronary artery lesions [18]. Serum specimens were collected from eligible patients for the detection of SIRT6 levels by enzyme-linked immunosorbent assay. Clinical data and the complete CAG inspection results of these participants were also collected.

### Ethical Considerations

The study protocol was approved by the human ethics committee of Ruijin Hospital (KY2021-108 Ruijin Hospital). It adhered to strict data confidentiality measures in compliance with the Helsinki Declaration and the institutional guidelines and reporting studies conducted using routinely collected observational data. Participants provided written informed consent at the time of data collection.

### Selection Bias

Several rigorous measures were implemented to address potential selection bias in this study. Stringent inclusion and exclusion criteria were carefully defined and applied to ensure a well-characterized and homogeneous study population. Furthermore, random sampling techniques were used where appropriate, and blinding in outcome assessment was ensured to minimize bias in both participant selection and data interpretation. These strategies were designed to enhance the internal validity and robustness of our study results.

### Data Collection

**Figure 1.**
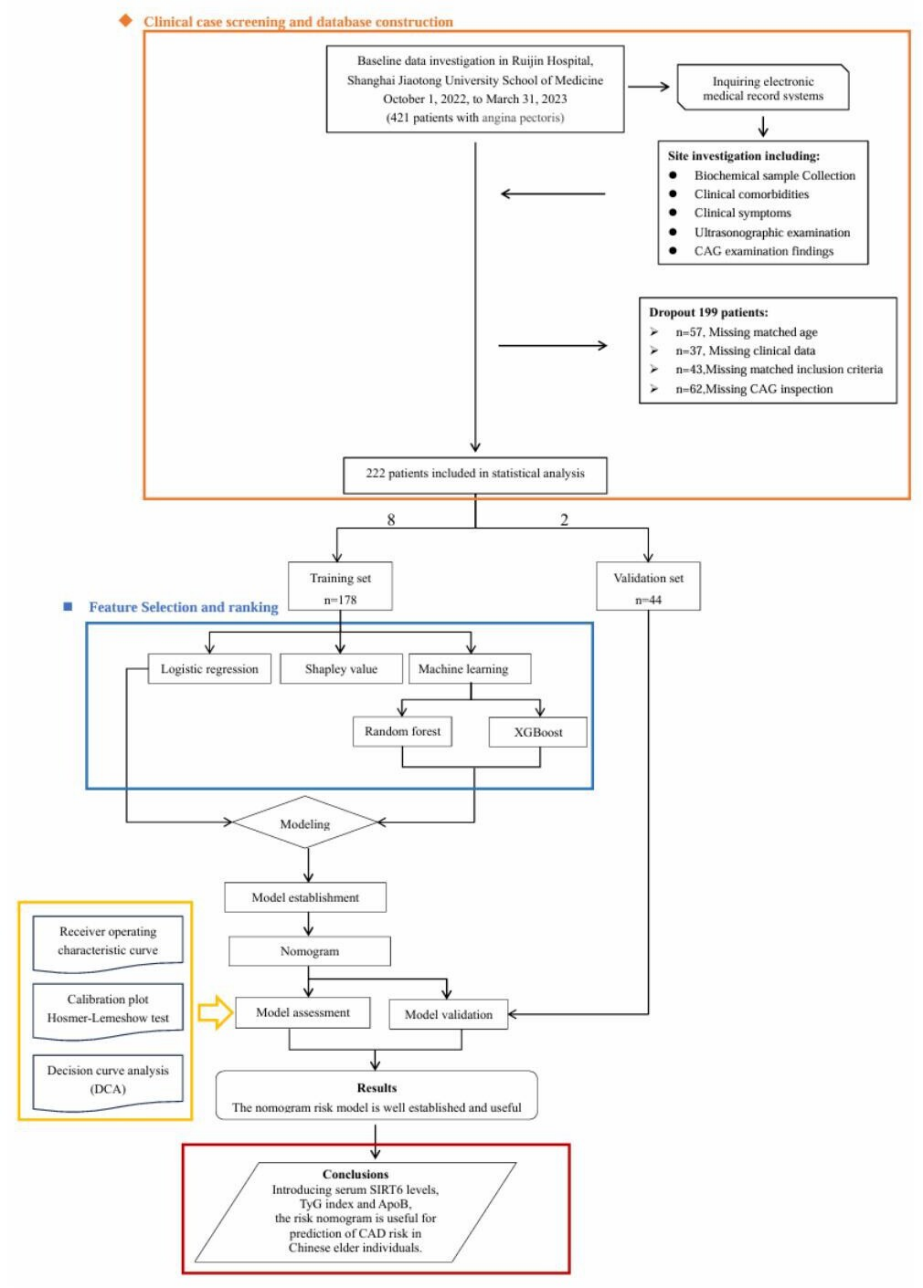
Study flowchart. ApoB: apolipoprotein B; CAD: coronary artery disease; CAG:coronary angiography; SIRT6: silent information regulator 6; TyG: triglyceride glucose; XGBoost: Extreme Gradient Boosting.

All recruited patients were divided into 2 cohorts, the development cohort and the validation cohort, in an approximate ratio of 8:2. Then, predefined criteria were applied to ensure cohort comparability. A total of 29 pretherapeutic parameters were collected, including demographic characteristics, initial symptoms, history of hypertension and diabetes mellitus, baseline clinical status, and baseline laboratory test results. Finally, 3 out of 26 collected parameters were tested as putative predictors for outcomes in the models. Three putative predictors allowed for 9-10 events per predictor for the primary outcome in the training cohort, meeting the recommended minimal number of events per predictor. These putative predictors were selected based on previous research demonstrating their potential prognostic value in CAD and the investigators’ (WFS, ZYC, and JWN) clinical experience with CAD. The study flowchart is shown in Figure 1.

### Statistical Analysis

#### Variable Selection

In this study, IBM SPSS Statistics (version 26.0; IBM Corp) software was used for baseline description and logistic regression (LR) analysis. Continuous variables were presented as mean (SD), while categorical variables were expressed as percentages (n [%]). Normality was assessed using the Shapiro-Wilk test, with *P*>.05 indicating adherence to normal distribution. For normally distributed variables, an intergroup analysis was conducted using the *t* test, while nonnormally distributed data were compared using the Wilcoxon rank sum test. Features with >30% missing values were removed. The remaining 29 features were collected for further processing. For features with missing values of <10%, median or mean imputation was used. Those with 10%‐20% missing values were imputed by the “MissForest” package in R (R Foundation for Statistical Computing). Random forest and Extreme Gradient Boosting (XGBoost) algorithms were implemented via R statistical software (version 4.3.2; R Foundation for Statistical Computing). Receiver operating characteristic (ROC) curves were plotted using the “pROC” package, while nomograms were constructed using the “rms” and “regplot” packages. The “val.prob” function was used for refining calibration curve plots and Hosmer-Lemeshow tests, while the “dcurves” function facilitated decision curve analysis (DCA). All statistical tests were 2-tailed, with *P*<.05 considered statistically significant. Feature ranking was obtained using Shapley Additive Explanation, Gini, and Gain values, respectively. A 10-fold cross-validation strategy was applied to develop the data set into training, validation, and test sets.

#### Model Building and Visualization:

Three methodologies, including LR, random forest, and XGBoost, were used for model building. All analyses were conducted using the statistical software package R (version 4.3.2). The data from the Ruijin Hospital of Shanghai Jiao Tong Medical University were used as the training set and internal validation set for model development and verification. Binary LR, random forest, and XGBoost models were constructed using the training data to classify patients into those with CAS and those with CAD. The XGBoost model exhibited the highest comprehensive discriminant ability [19] and therefore was selected for further analysis. The final model was visualized as a column line chart to address issues of poor machine learning interpretability and consequent low clinical use.

#### Model Comparison

Accuracy, precision, recall, and *F*_1_-score were used to evaluate the performance of the multiclassification model. The model error was further analyzed using a confusion matrix. Discrimination, calibration, and clinical use between the nomogram and the variables incorporated into the nomogram were assessed in both the training and validation sets, respectively. ROC curves, calibration curves, and DCA plots were plotted, and the areas under the ROC curves (AUROCs) were compared using the Delong test. Calibration was evaluated using the Hosmer-Lemeshow test.

## Results

### Characteristics of the Training and Validation Sets

The final study cohort comprised 222 patients, with 178 allocated to the training set and 44 to the validation set. The demographic characteristics and clinical results of patients in both sets are summarized in Table 1. There were 23.87% (53/222) of patients in the CAS group and 76.13% (169/222) of patients in the CAD group. There were significant differences in the low-density lipoprotein cholesterol (LDL-C), apolipoprotein B (ApoB), apolipoprotein E (ApoE), fasting blood glucose (FBG), hemoglobin A_1c_ (HbA_1c_), Gensini score, TyG index, and atherogenic index of plasma (AIP) between the CAS and CAD groups (*P*<.05). Specifically, the CAD group exhibited a higher TyG index (12.4 [SD 1.38]) and AIP (0.10 [SD 0.31]) than the CAS group (10.9 [SD 0.54], 0.00 [SD 0.27]), as shown in Table 1. Significant differences were also observed in categorical variables, such as sex, history of diabetes, clinical symptoms, segmental vascular lesions (ie, LAD, left circumflex artery, left main coronary artery, and right coronary artery), and 10-year cardiovascular risk between the CAS and CAD groups (*P*<.05).

**Table 1.** Baseline characteristics of participants^a^.

Variables	CAS^b^ (n=53)	CAD^c^ (n=169)	*P* value
Age (years), mean (SD)	69.3 (5.33)	68.9 (5.12)	.56
**Sex, n (%)**			.03*
Female	28 (52.8)	59 (34.9)	
Male	25 (47.2)	110 (65.1)	
BMI, mean (SD)	25.1 (4.46)	24.6 (2.64)	.50
**Drinking, n (%)**			1.00
No	47 (88.7)	151 (89.3)	
Yes	6 (11.3)	18 (10.7)	
**Smoking, n (%)**			.68
No	47 (88.7)	144 (85.2)	
Yes	6 (11.3)	25 (14.8)	
SBP^d^, mean (SD)	145 (18.3)	146 (17.8)	.82
DBP^e^, mean (SD)	77.6 (10.1)	78.5 (10.8)	.60
**Diabetes mellitus, n (%)**			.006**
No	43 (81.1)	100 (59.2)	
Yes	10 (18.9)	69 (40.8)	
**Hypertension, n (%)**			.65
No	20 (37.7)	56 (33.1)	
Yes	33 (62.3)	113 (66.9)	
hs-CRP^f^, mean (SD)	2.19 (3.37)	2.15 (3.32)	.94
CysC^g^, mean (SD)	1.00 (0.16)	1.59 (7.08)	.28
eGFR^h^, mean (SD)	84.1 (9.63)	82.9 (10.6)	.47
mALB^i^, mean (SD)	1.40 (1.78)	2.03 (5.04)	.17
cTnI^j^, mean (SD)	7.60 (16.1)	25.3 (114)	.05
proBNP^k^, mean (SD)	170 (338)	305 (1764)	.35
LDL-C^l^, mean (SD)	2.36 (0.89)	1.95 (0.69)	.003**
Lp(a)^m^, mean (SD)	0.26 (0.32)	0.31 (0.35)	.35
sLDL^n^, mean (SD)	0.76 (0.35)	0.66 (0.31)	.08
ApoA1^o^, mean (SD)	1.35 (0.25)	1.28 (0.24)	.10
ApoB^p^, mean (SD)	0.76 (0.23)	0.68 (0.19)	.02*
ApoE^q^, mean (SD)	3.77 (0.52)	3.60 (0.62)	.05
FBG^r^, mean (SD)	5.67 (1.00)	6.10 (1.72)	.03*
HbA_1c_^s^, mean (SD)	6.00 (0.66)	6.46 (1.28)	.001**
FINS^t^, mean (SD)	9.87 (5.37)	11.7 (11.3)	.11
CP^u^, mean (SD)	2.61 (1.16)	2.72 (1.23)	.56
**Carotid plaque, n (%)**			.049*
<50%	37 (69.8)	138 (81.7)	
>50%	1 (1.89)	9 (5.33)	
No	15 (28.3)	22 (13)	
**Ten-year cardiovascular risk, n (%)**			.009**
High	21 (39.6)	107 (63.3)	
Low	7 (13.2)	15 (8.88)	
Middle	25 (47.2)	47 (27.8)	
**LAD** ^v^ **, n (%)**			.01*
No	11 (20.8)	12 (7.10)	
Yes	42 (79.2)	157 (92.9)	
**RCA** ^w^ **, n (%)**			<.001***
No	24 (45.3)	27 (16)	
Yes	29 (54.7)	142 (84)	
**LCX** ^x^ **, n (%)**			<.001***
No	38 (71.7)	57 (33.7)	
Yes	15 (28.3)	112 (66.3)	
**LM** ^y^ **, n (%)**			.01*
No	53 (100)	147 (87)	
Yes	N/A^z^	22 (13)	
SIRT6^aa^, mean (SD)	866 (510)	495 (443)	<.001***
TyG^ab^, mean (SD)	10.9 (0.54)	12.4 (1.38)	<.001***
AIP^ac^, mean (SD)	0.00 (0.27)	0.10 (0.31)	.02*

a Variables of significance (*P≤.05, ** *P*≤.01, and *** *P*≤.001).

b CAS, coronary atherosclerosis.

c CAD: coronary artery disease.

d SBP: systolic pressure.

e DBP: diastolic blood pressure.

f hs-CRP: hypersensitive C-reactive protein.

g CysC: cystatin c.

h eGFR: estimated glomerular filtration rate.

i mALB: microalbumin.

j cTnI: troponin I.

k proBNP: pro-B-type natriuretic peptide.

l LDL-C: low-density lipoprotein cholesterol.

m Lp(a): lipoprotein(a).

n sLDL: small dense low-density lipoprotein.

o ApoA1: apolipoprotein A1.

p ApoB: apolipoprotein B.

q ApoE: apolipoprotein E.

r FBG: fasting blood glucose.

s HbA_1c_: hemoglobin A_1c_.

t FINS: fasting insulin.

u CP: C-peptide.

v LAD: left anterior descending branch.

w RCA: right coronary artery.

x LCX: left circumflex artery.

y LM: left main coronary artery.

z N/A: not applicable.

aa SIRT6: silent information regulator 6.

ab TyG: triglyceride glucose.

ac AIP: atherogenic index of plasma.

### Circulating SIRT6 Levels in Patients

**Figure 2.**
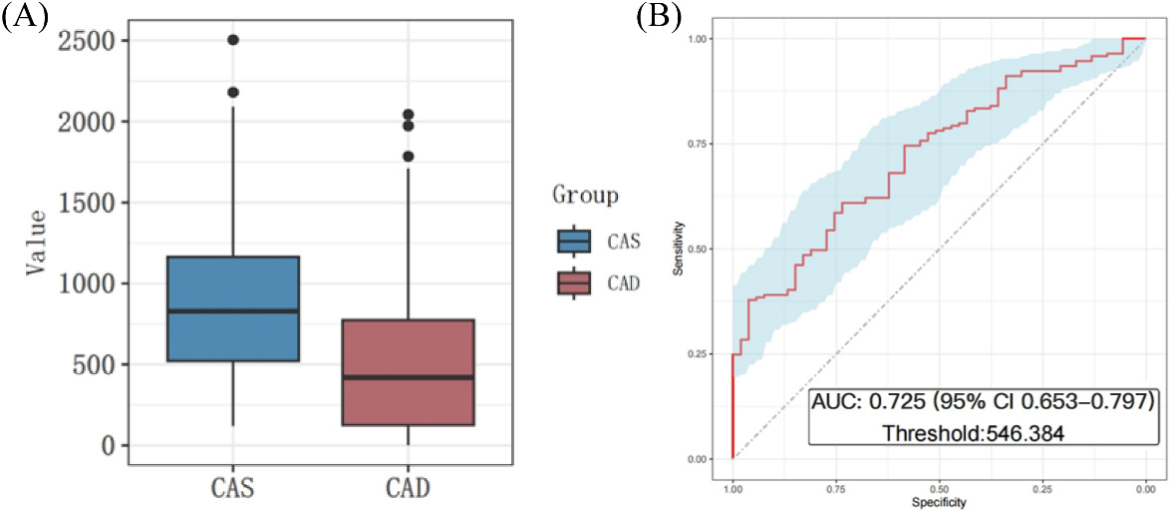
Serum levels of silent information regulator 6 (SIRT6) and receiver operating characteristic (ROC) curve. (**A**) Serum levels of SIRT6 were lower in patients with CAD and patients with CAS. (**B**) ROC curve analysis of SIRT6 in predicting the diagnosis of CAD (AUROC 0.725, 95% CI 0.653‐0.797). AUROC: area under the receiver operating characteristic curve; CAD: coronary artery disease; CAS: coronary atherosclerosis.

To investigate the potential difference in serum SIRT6 levels between the CAD and CAS groups, the enzyme-linked immunosorbent assay was used to determine the SIRT6 levels in serum samples. A significant reduction in serum SIRT6 levels was observed in the CAD group (495 [SD 443] pg/mL) compared with the CAS group (866 [SD 510] pg/mL) (Figure 2A). To further assess the diagnostic potential of serum SIRT6 as a biomarker for distinguishing between CAS and CAD, ROC analysis was performed. A serum SIRT6 level of 546.384 pg/mL was identified as the optimal cutoff value for discriminating between CAS and CAD (AUROC 0.725, 95% CI 0.653‐0.797) (Figure 2B).

### Exploration of Variable Correlations

**Figure 3.**
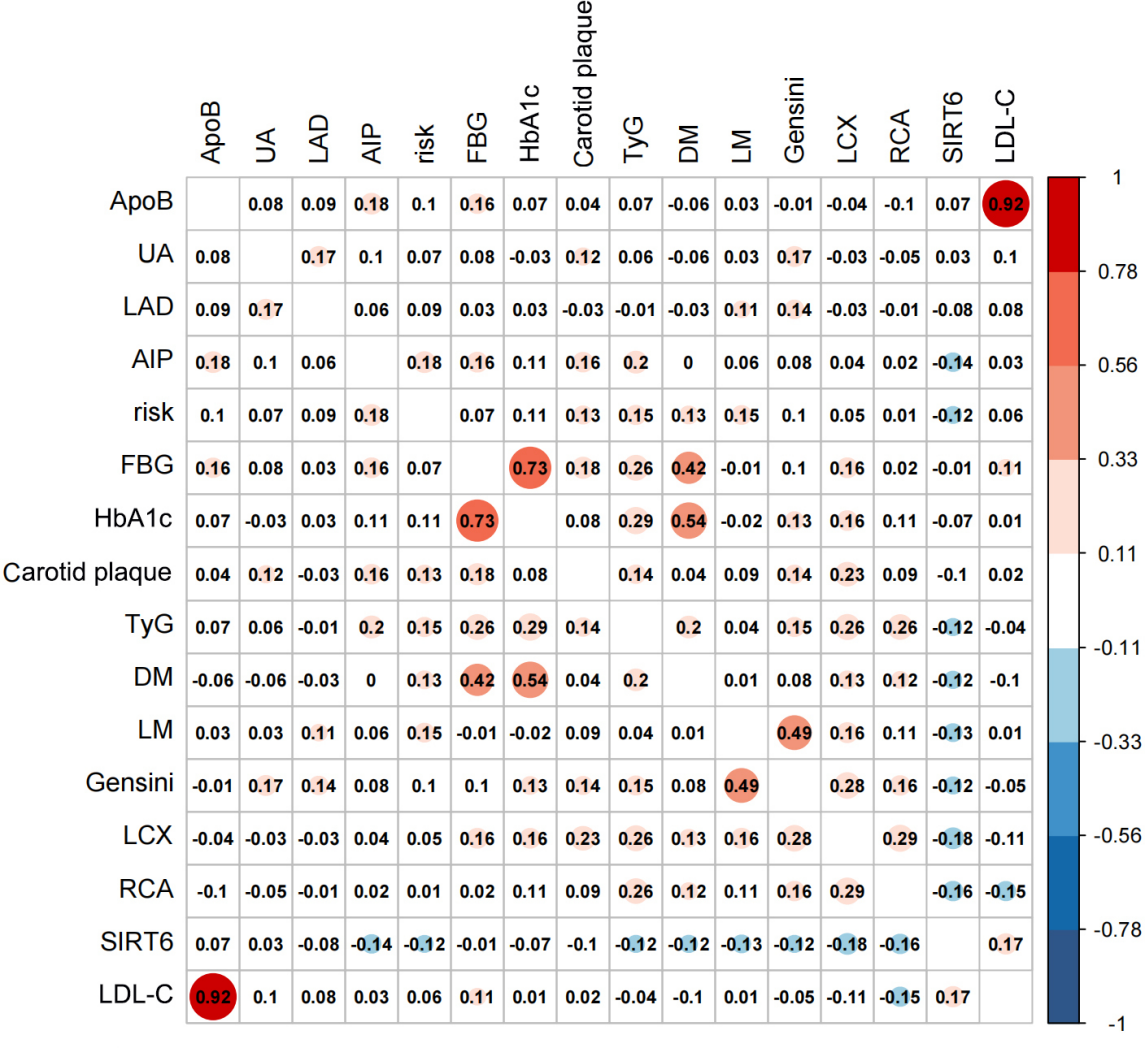
Correlation heatmap analysis. AIP: atherogenic index of plasma; ApoB: apolipoprotein B; DM: diabetic mellites; FBG: fasting blood glucose; HbA_1c_: hemoglobin A_1c_; LAD: left anterior descending branch; LCX: left circumflex artery; LDL-C: low-density lipoprotein cholesterol; LM: left main coronary artery; RCA: right coronary artery; Risk: 10-year cardiovascular risk; SIRT6: silent information regulator 6; TyG: triglyceride glucose; UA: unstable angina pectoris.

Factors with significant differences in intergroup analysis, including ApoB, unstable angina pectoris, LDL-C, AIP, FBG, HBA_1c_, TyG index, history of diabetes mellitus, SIRT6, LAD, AIP, 10-year cardiovascular risk, carotid plaque burden, segmental vascular lesions, and Gensini score, were selected for further interfactor correlation analysis. To illustrate the correlations among these predictive indicators, a correlation coefficient matrix graph was constructed. While serum SIRT6 did not exhibit positive correlations with other indicators, aside from LDL-C, it demonstrated negative correlations with the Gensini score, 10-year cardiovascular risk, and carotid plaque burden (Figure 3).

A correlation coefficient matrix graph was constructed. Darker shades represent stronger correlations, with positive correlations indicated by positive values and negative correlations by negative values. The predictive indicators included in this analysis are primarily independent predictive factors.

### Feature Selection and Ranking

**Figure 4.**
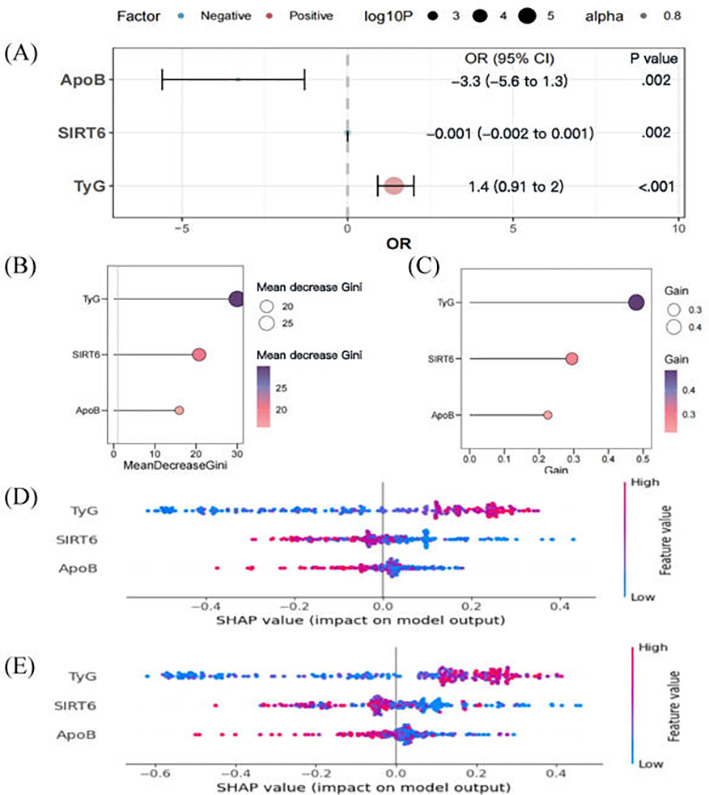
Feature importance. (**A**) Logistic regression results; (**B**) random forest results; (**C**) XGBoost results; (**D**) random forest SHAP; and (**E**) XGBoost SHAP. ApoB: apolipoprotein B; OR: odds ratio; SHAP: Shapley Additive Explanation; SIRT6: silent information regulator 6; TyG: triglyceride glucose; Extreme Gradient Boosting XGBoost.

The significance of variables was preliminarily evaluated by LR analysis. Ultimately, serum SIRT6 levels, TyG index, and ApoB were incorporated into the model construction (Figure 4A). Feature importance ranking was determined using Gini (Figure 4B), Gain (Figure 4C), and Shapley Additive Explanation values (Figure 4D and E), which yielded similar results (Figure 4).

### Model Performance Comparisons

**Figure 5.**
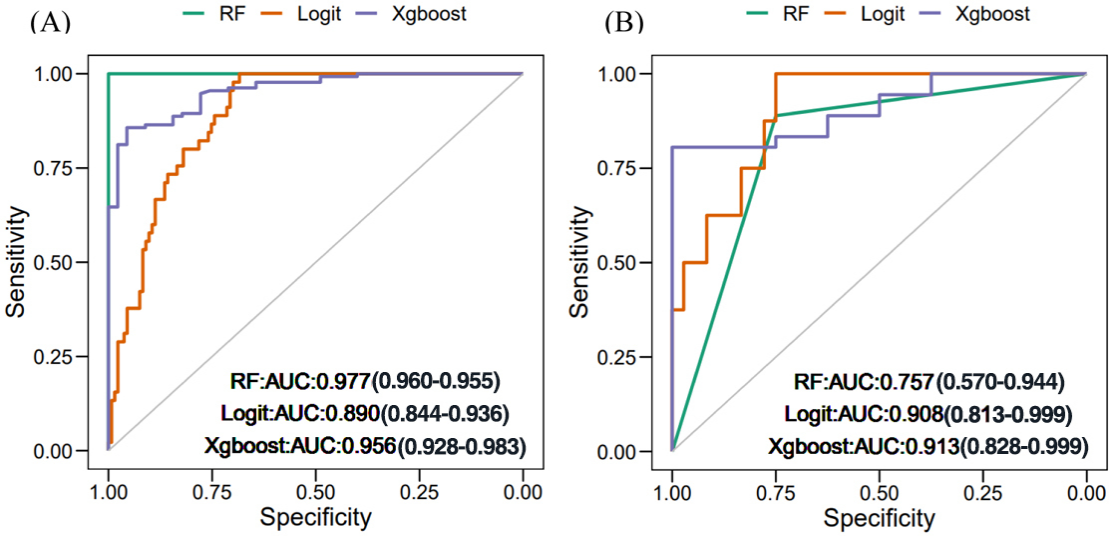
Receiver operating characteristic curves for predicting different classes using various models. (**A**) AUROC of the training set and (**B**) AUROC of the validation set. AUROC: area under the receiver operating characteristic curve; RF: random forest; Xgboost: Extreme Gradient Boosting.

An LR model and 2 machine learning models were constructed to predict the development of CAD in older adult patients. The discriminative performance of the 3 models is shown via ROC curves in Figure 5.

In the training set, the random forest model demonstrated the best predictive ability for CAD in older adult patients (AUROC 0.977, 95% CI 0.960‐0.995), followed by the XGBoost (AUROC 0.956) and LR (AUROC 0.890) models (Figure 5A). However, in the validation set, the classification performance of the random forest model was noticeably inferior to that of the XGBoost model. The AUROC values of XGBoost, LR, and random forest models were 0.913, 0.908, and 0.757, respectively (Figure 5B).

**Figure 6.**
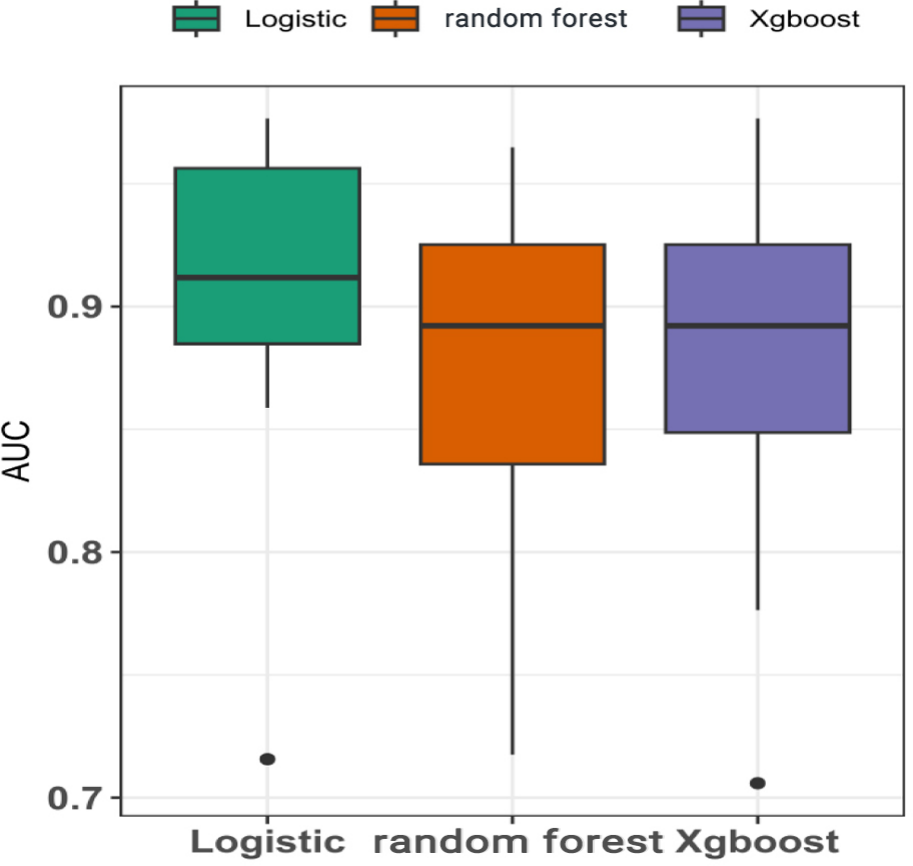
Cross-validation AUROC of various models. AUROC: area under the receiver operating characteristic curve; Xgboost: Extreme Gradient Boosting.

In cross-validation, the LR model demonstrated the best predictive ability for CAD in older adult patients (AUROC 0.912), followed by the XGBoost (AUROC 0.892) and random forest (AUROC 0.892) models (Figure 6).

**Figure 7.**
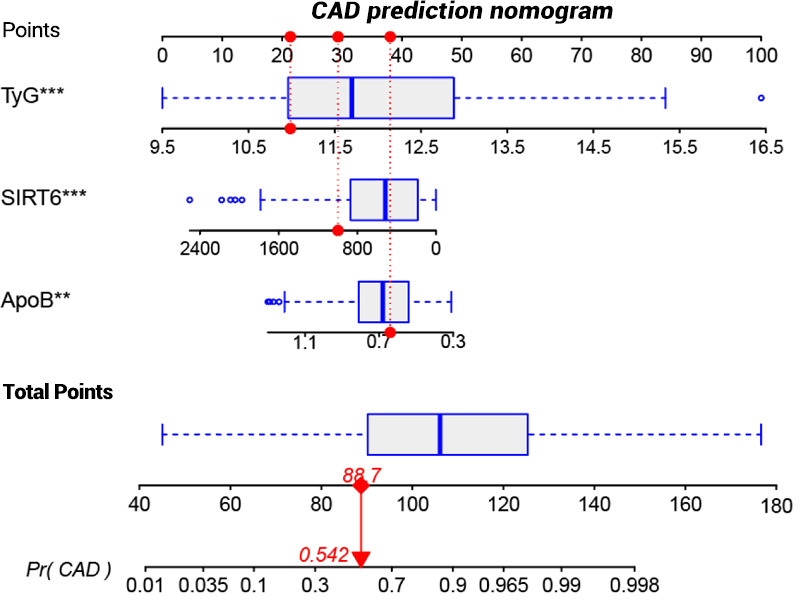
Nomogram for predicting the probability of CAD. Variables of significance (**P≤.01 and ***P≤.001). ApoB: apolipoprotein B; CAD: coronary artery disease; SIRT6: silent information regulator 6; TyG: triglyceride glucose.

Detailed performance metrics of the 3 models are shown in Table 2. The XGBoost model exhibited the best discrimination, with the highest recall (1) and accuracy (0.863), the second-highest precision (0.833), and the second-highest *F*_1_-score (0.909). Although the XGBoost model may not perform as well as the other 2 models in certain aspects, its overall stability and balanced performance on both the training and the validation sets make it a more reliable choice. As a results, we developed a CAD prediction model for the older adults using the XGBoost algorithm and visualized the results through a nomogram. The nomogram based on the serum SIRT6-level model is shown in Figure 7.

**Table 2.** Model performance metrics.

Model	Accuracy	Precision	Recall	*F*_1_-score
LR^a^	0.795	0.75	1	0.857
RF^b^	0.863	0.941	0.889	0.914
XGBoost^c^	0.863	0.833	1	0.909

a LR: logistic regression.

b RF: random forest.

c XGBoost: Extreme Gradient Boosting.

**Figure 8.**
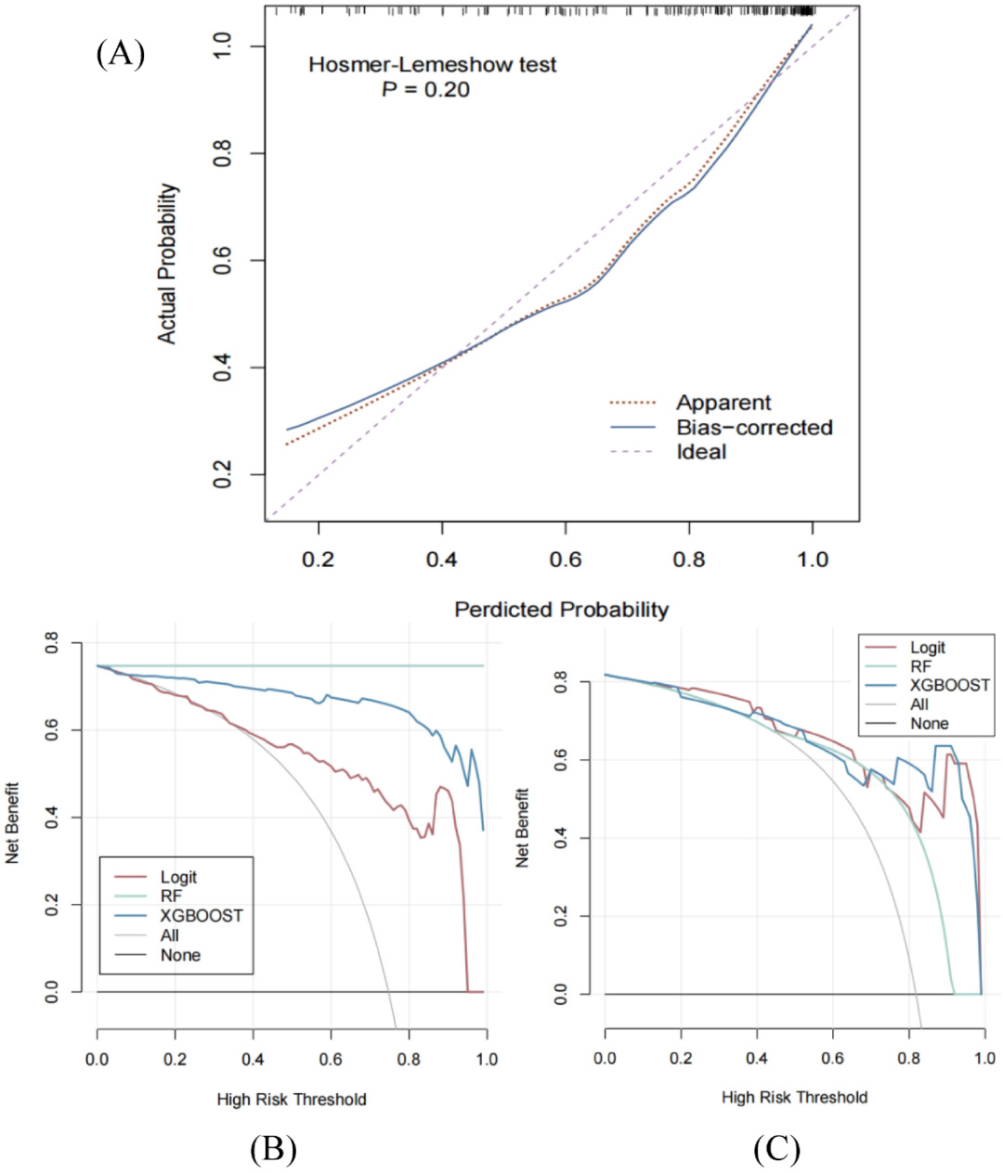
Calibration curve and decision curve analyses for predicting coronary artery disease. (**A**) Calibration curves of the training cohort; (**B**) decision curve analysis (DCA) of the training cohort; and (**C**) DCA of the validation cohort. RF: random forest; XGBoost: Extreme Gradient Boosting.

The calibration curves (Figure 8A) showed a similar trend among the 3 models, and the Hosmer-Lemeshow test results (*χ*^2_8_^=11.001; *P*=.20) indicated no significant difference between the predicted and observed values. These data suggest that the XGBoost model has good calibration ability.

The DCA revealed that the XGBoost model exhibited greater net benefit along with the threshold probability, indicating its superior clinical use compared with other models (Figure 8B and C).

## Discussion

### Principal Findings

An accurate risk assessment is critical for patient-centered clinical decision-making. The nomogram presented in this study may serve as an important clinical decision aid tool, assisting in determining whether invasive CAG should be performed, or informing patients as a basis for joint decision-making. In this study, traditional LR and 2 common machine learning algorithms were used to establish a prediction and evaluation model of coronary artery stenosis in older adult patients with suspected CAD. Through a comprehensive analysis of models, the XGBoost algorithm–based model with excellent prediction performance and good internal verification ability was selected. In addition, we developed a nomogram for CAD prediction by combining traditional clinical variables (eg, ApoB) with 2 novel variables relevant to the older adult population, serum SIRT6 levels and the TyG index, which allowed for assessing the risk of coronary artery stenosis more conveniently and interactively (Figure 6). This, to some extent, helps address the issue of poor interpretability, which often hampers the implementation of machine learning models.

Given its significant role in genomic stability, DNA repair, and telomere function, SIRT6 has been considered to have therapeutic potential in aging and aging-related diseases. Moreover, SIRT6 plays a crucial role in the development and progression of CAD due to its involvement in oxidative stress, inflammation, and energy metabolism [20]. Previous studies have highlighted the importance of SIRT6 in protecting blood vessels and the heart from endothelial dysfunction, atherosclerosis, myocardial fibrosis, and ischemia or reperfusion injury [21,22].

In this study, we observed lower serum SIRT6 levels in older adult patients with CAD than in those with CAS. Correlation analysis revealed a negative association between serum SIRT6 levels and the Gensini score, suggesting that reduced serum SIRT6 levels may exacerbate the severity of coronary artery lesions. Furthermore, serum SIRT6 levels were negatively correlated with segmental vascular lesions and carotid plaque burden, indicating that serum SIRT6 may reflect the overall burden of atherosclerotic plaques in blood vessels. Dyslipidemia is an independent risk factor for the occurrence and development of CAD, while diabetes mellitus doubles the risk of cardiovascular disease and is a leading cause of mortality in patients with dyslipidemia [23,24].

LDL-C levels are considered a determinant of the absolute risk of major cardiovascular events [25]. Previous evidence has revealed that liver-specific knockout of SIRT6 significantly increases the proprotein convertase subtilisin/kexin type 9 (*PCSK9*) gene expression and plasma LDL-C levels, while SIRT6 overexpression improves lipid metabolism and reduces atherosclerosis risk by lowering plasma LDL-C and *PCSK9* levels [26]. Although a previous clinical study in a Chinese population of all ages found no correlation between serum SIRT6 and LDL-C levels [15], in this study, we found a positive correlation between serum SIRT6 and LDL-C levels. Future investigations are needed to explore the mechanisms by which SIRT6 affects cardiovascular function through lipid metabolism in the older adult population.

Disruption of glucose homeostasis is another recognized risk factor for CAD. Animal models have shown that SIRT6 plays an important role in glucose production and uptake, insulin signaling, and metabolism [27]. Our data revealed that circulating SIRT6 levels were negatively correlated with FBG and the comorbidity of diabetes, which is consistent with previous findings [28]. Whether glucose metabolism disruption mediates the relationship between SIRT6 and CAD warrants further investigation. In addition, our study identified serum SIRT6 as an independent biomarker for assessing the severity of coronary artery lesions in older adults, with the optimal cutoff value for classifying atherosclerosis and CAD being 546.384 pg/mL.

Abnormal blood lipids are established independent risk factors for CAD. Unlike previous studies that commonly included LDL-C, HDL-C, and other conventional measures, our study incorporated ApoB as one of the predictive factors. ApoB is a crucial component of LDL-C, and its deposition within the arterial wall is a fundamental step driving the progression of atherosclerosis, from initial lipid deposition to the development of acute complex events, such as plaque rupture [29]. Compared with other individual lipid markers, ApoB serves as a more accurate biomarker for cardiovascular risk assessment [30,31]. Elevated ApoB levels, representing LDL levels, are associated with the occurrence of CAD, and ApoB levels are positively correlated with the Gensini score, indicating the degree of atherosclerosis and arterial narrowing [32]. In addition, circulating ApoB levels are considered more predictive than plasma cholesterol levels for early-onset CAD risk [33]. Correlation analysis indicated a significant positive correlation between ApoB and LDL-C levels. Moreover, in all 3 statistical methods used for feature selection, ApoB consistently emerged as significant, which is in line with previous findings [31]. However, in an intergroup analysis, we observed higher levels of ApoB in the CAS group than in the CAD group, which is contrary to previous results [33].

Insulin resistance further disturbs glucose and lipid metabolism in patients with diabetes, promoting chronic inflammation, disrupting normal endothelial function, and accelerating the development of complications, such as atherosclerosis-related cardiovascular diseases. The persistent prevalence of insulin resistance is considered a major contributor to the high mortality rate in atherosclerosis-related cardiovascular diseases worldwide [34]. The TyG index is a quantitative measure based on blood glucose and triglycerides that assesses insulin resistance. A clinical study has shown an association between the TyG index and coronary artery calcification [35]. However, in cohort studies, the TyG index was found to be correlated with carotid atherosclerosis [36] but unrelated to the incidence of CAD [37]. In our study, intergroup analysis revealed that the TyG index was significantly higher in the CAD group than in the CAS group. Correlation analysis indicated positive associations between the TyG index and the Gensini score, carotid plaque burden, and segmental vascular lesions. Furthermore, in multifactorial regression analysis and machine learning feature selection, the TyG index consistently demonstrated a strong correlation with CAD, suggesting that it may be an independent risk factor for CAD. Furthermore, we observed a negative correlation between serum SIRT6 levels and the TyG index. Future research is needed to explore the impact of SIRT6 on the TyG index and to elucidate the relationship between these factors in terms of glucose and lipid metabolism disturbance and the development of CAD.

### Strengths and Limitations

Our prediction model addresses the challenge of poor interpretability associated with machine learning models. By incorporating readily obtainable clinical and physiological indicators, this nomogram not only provides prediction results and probabilities directly but also facilitates personalized intervention through probability curves. Tailored patient treatment may reduce medical costs and unnecessary invasive tests. Particularly in resource-constrained medical settings, our model may assist in disease screening and alleviate the medical burden.

Although the overall performance of the model was good, the sample distribution in our study was not balanced, and the model was not validated externally with independent data sets. Therefore, the generalizability and extrapolation to the overall population cannot be currently estimated. Second, the model was more inclined to classify patients without the disease as having the disease, while the proportion of patients with the disease misclassified as being disease-free was very low. The rate of missed diagnosis was also low. Third, the limited small sample size and single-center, cross-sectional design constrained the generalizability of the results. Furthermore, this study specifically focused on the older adult population; however, there is currently no consensus on age-related changes in circulating ApoB levels. Furthermore, some studies have suggested that the ApoB–apolipoprotein A1 (ApoA1) ratio has a higher predictive value for atherosclerosis and intima-media thickness than individual lipid markers. Therefore, in future research, expanding the sample size and using the ApoB-ApoA1 ratio as a composite indicator are needed to further evaluate the correlation between ApoB and the severity of coronary artery lesions.

### Conclusions

Based on the LR, random forest, and XGBoost algorithms, we developed a predictive model for CAD occurrence in the older adult population, incorporating SIRT6, the TyG index, and ApoB. The model was comprehensively evaluated for discrimination, calibration, clinical applicability, and internal validation. The XGBoost predictive model exhibited favorable predictive performance and clinical use, which may facilitate early CAD screening and diagnosis in older adult patients, particularly for identifying high-risk individuals. Furthermore, the model may reduce unnecessary invasive examinations in negative patients and minimize missed diagnoses in positive patients. The personalized probability curve generated by the model may offer targeted intervention guidance. Our findings also underscore the importance of considering risk factors such as SIRT6, ApoB, and TyG levels in this population.

## Abbreviations

AIP: atherogenic index of plasma
ApoA1: apolipoprotein A1
ApoB: apolipoprotein B
ApoE: apolipoprotein E
AUROC: area under the receiver operating characteristic curve
CAD: coronary artery disease
CAG: coronary angiography
CAS: coronary atherosclerosis
DCA: decision curve analysis
FBG: fasting blood glucose
HbA_1c_: hemoglobin A_1c_
LAD: left anterior descending branch
LDL-C: low-density lipoprotein cholesterol
LR: logistic regression
*PCSK9*: proprotein convertase subtilisin/kexin type 9
ROC: receiver operating characteristic
SIRT6: silent information regulator 6
TyG: triglyceride glucose
XGBoost: Extreme Gradient Boosting

## References

[1] Mensah GA, Fuster V, Murray CJL, Roth GA, Global Burden of Cardiovascular Diseases and Risks Collaborators (2023). Global burden of cardiovascular diseases and risks, 1990-2022. J Am Coll Cardiol.

[2] Crea F (2022). The burden of cardiovascular risk factors: a global perspective. Eur Heart J.

[3] Liu S, Li Y, Zeng X (2019). Burden of cardiovascular diseases in China, 1990-2016: findings from the 2016 Global Burden of Disease Study. JAMA Cardiol.

[4] Roth GA, Mensah GA, Fuster V (2020). The global burden of cardiovascular diseases and risks: a compass for global action. J Am Coll Cardiol.

[5] Goyal P, Kwak MJ, Al Malouf C (2022). Geriatric cardiology: coming of age. JACC Adv.

[6] Wan X, Ren H, Ma E, Yang G (2017). Mortality trends for ischemic heart disease in China: an analysis of 102 continuous disease surveillance points from 1991 to 2009. BMC Public Health.

[7] The Lancet (2022). Population ageing in China: crisis or opportunity?. Lancet.

[8] He X, Song M, Qu J (2019). Basic and translational aging research in China: present and future. Protein Cell.

[9] Bjarnason-Wehrens B (2018). Elderly patients with ischaemic heart disease need our attention!. Eur J Prev Cardiol.

[10] Nanna MG, Chen ST, Nelson AJ, Navar AM, Peterson ED (2020). Representation of older adults in cardiovascular disease trials since the inclusion across the lifespan policy. JAMA Intern Med.

[11] Forman DE, Maurer MS, Boyd C (2018). Multimorbidity in older adults with cardiovascular disease. J Am Coll Cardiol.

[12] Mendieta G, Pocock S, Mass V (2023). Determinants of progression and regression of subclinical atherosclerosis over 6 years. J Am Coll Cardiol.

[13] Cho SMJ, Koyama S, Honigberg MC (2023). Genetic, sociodemographic, lifestyle, and clinical risk factors of recurrent coronary artery disease events: a population-based cohort study. Eur Heart J.

[14] Guo Z, Li P, Ge J, Li H (2022). SIRT6 in aging, metabolism, inflammation and cardiovascular diseases. Aging Dis.

[15] Yan Z, Wang X, Liu YS, Xing XW, Zhang XG, Lu QH (2021). Decreased serum SIRT6 as a novel predictor of coronary artery disease. Eur Rev Med Pharmacol Sci.

[16] Piccininni M, Rohmann JL, Huscher D (2020). Performance of risk prediction scores for cardiovascular mortality in older persons: external validation of the SCORE OP and appraisal. PLoS One.

[17] Rao SV, Stone GW (2016). Arterial access and arteriotomy site closure devices. Nat Rev Cardiol.

[18] Rampidis GP, Benetos G, Benz DC, Giannopoulos AA, Buechel RR (2019). A guide for Gensini Score calculation. Atherosclerosis.

[19] Han JW, Lee SK, Kwon JH (2024). A machine learning algorithm facilitates prognosis prediction and treatment selection for Barcelona clinic liver cancer stage C hepatocellular carcinoma. Clin Cancer Res.

[20] Guo J, Wang Z, Wu J (2019). Endothelial SIRT6 is vital to prevent hypertension and associated cardiorenal injury through targeting Nkx3.2-GATA5 signaling. Circ Res.

[21] Zhu Y, Hu S, Pan X (2023). Hepatocyte Sirtuin 6 protects against atherosclerosis and steatohepatitis by regulating lipid homeostasis. Cells.

[22] Zhang Q, Tu W, Tian K (2019). Sirtuin 6 inhibits myofibroblast differentiation via inactivating transforming growth factor‐β1/Smad2 and nuclear factor‐κB signaling pathways in human fetal lung fibroblasts. J of Cell Biochem.

[23] Pastromas S, Terzi AB, Tousoulis D, Koulouris S (2008). Postprandial lipemia: an under-recognized atherogenic factor in patients with diabetes mellitus. Int J Cardiol.

[24] McAuley PA, Artero EG, Sui X, Lavie CJ, Almeida MJ, Blair SN (2014). Fitness, fatness, and survival in adults with prediabetes. Diabetes Care.

[25] Ference BA, Graham I, Tokgozoglu L, Catapano AL (2018). Impact of lipids on cardiovascular health: JACC health promotion series. J Am Coll Cardiol.

[26] Tao R, Xiong X, DePinho RA, Deng CX, Dong XC (2013). FoxO3 transcription factor and Sirt6 deacetylase regulate low density lipoprotein (LDL)-cholesterol homeostasis via control of the proprotein convertase subtilisin/kexin type 9 (Pcsk9) gene expression. J Biol Chem.

[27] Mostoslavsky R, Chua KF, Lombard DB (2006). Genomic instability and aging-like phenotype in the absence of mammalian SIRT6. Cell.

[28] Wu X, Liu H, Brooks A (2022). SIRT6 mitigates heart failure with preserved ejection fraction in diabetes. Circ Res.

[29] Borén J, Williams KJ (2016). The central role of arterial retention of cholesterol-rich apolipoprotein-B-containing lipoproteins in the pathogenesis of atherosclerosis: a triumph of simplicity. Curr Opin Lipidol.

[30] Ference BA, Kastelein JJP, Ray KK (2019). Association of triglyceride-lowering LPL variants and LDL-C-lowering LDLR variants with risk of coronary heart disease. JAMA.

[31] Welsh C, Celis-Morales CA, Brown R (2019). Comparison of conventional lipoprotein tests and apolipoproteins in the prediction of cardiovascular disease. Circulation.

[32] Campeau L, Enjalbert M, Lespérance J (1984). The relation of risk factors to the development of atherosclerosis in saphenous-vein bypass grafts and the progression of disease in the native circulation. A study 10 years after aortocoronary bypass surgery. N Engl J Med.

[33] Quispe R, Martin SS, Michos ED (2021). Remnant cholesterol predicts cardiovascular disease beyond LDL and ApoB: a primary prevention study. Eur Heart J.

[34] Bornfeldt KE, Tabas I (2011). Insulin resistance, hyperglycemia, and atherosclerosis. Cell Metab.

[35] Lee SB, Ahn CW, Lee BK (2018). Association between triglyceride glucose index and arterial stiffness in Korean adults. Cardiovasc Diabetol.

[36] Irace C, Carallo C, Scavelli FB (2013). Markers of insulin resistance and carotid atherosclerosis. A comparison of the homeostasis model assessment and triglyceride glucose index. Int J Clin Pract.

[37] Vega GL, Barlow CE, Grundy SM, Leonard D, DeFina LF (2014). Triglyceride-to-high-density-lipoprotein-cholesterol ratio is an index of heart disease mortality and of incidence of type 2 diabetes mellitus in men. J Investig Med.

